# Human Germline Mutation and the Erratic Evolutionary Clock

**DOI:** 10.1371/journal.pbio.2000744

**Published:** 2016-10-19

**Authors:** Priya Moorjani, Ziyue Gao, Molly Przeworski

**Affiliations:** 1 Department of Biological Sciences, Columbia University, New York, New York, United States of America; 2 Howard Hughes Medical Institute & Dept. of Genetics, Stanford University, Stanford, California, United States of America; 3 Department of Systems Biology, Columbia University, New York, New York, United States of America

## Abstract

Our understanding of the chronology of human evolution relies on the “molecular clock” provided by the steady accumulation of substitutions on an evolutionary lineage. Recent analyses of human pedigrees have called this understanding into question by revealing unexpectedly low germline mutation rates, which imply that substitutions accrue more slowly than previously believed. Translating mutation rates estimated from pedigrees into substitution rates is not as straightforward as it may seem, however. We dissect the steps involved, emphasizing that dating evolutionary events requires not “a mutation rate” but a precise characterization of how mutations accumulate in development in males and females—knowledge that remains elusive.

## Introduction

One of the most fundamental discoveries in evolutionary biology is the “molecular clock”: the observation that changes to the genome along an evolutionary lineage accumulate steadily with time [1–3] and the subsequent development of a theory—the Neutral Theory—that explains why this behavior is expected for neutral genetic changes (i.e., changes with no fitness effects) [4,5]. We now understand that neutral mutations fix in the population at the rate at which they arise, irrespective of demographic history or natural selection at linked sites [4,6]. Thus, the accumulation of neutral substitutions over generations provides a record of the time elapsed on a lineage. It is this “evolutionary clock” that allows researchers to date past events.

Conversely, the existence of an evolutionary clock allows the number of substitutions on a lineage to be translated into a yearly mutation rate, given an independent estimate of when that lineage branched off [2,7–9]. For example, interpreting the fossil record as reflecting a 30 million year (My) split time between humans (apes) and rhesus macaques (Old World Monkey [OWM]) and using the average nucleotide divergence of ~6.2% between the two species [10] suggests an average yearly mutation rate of 10^−9^ per base pair (bp). Until 2010, single nucleotide substitutions were the main source of data from which to learn about mutation rates, and analyses of substitution patterns consistently suggested rates of around 10^−9^ per bp per year for primates [9,11–13].

Recent findings in human genetics therefore threw a spanner in the works when they suggested de novo mutation rates estimated from human pedigrees to be less than half what was previously believed, or approximately 0.5 x 10^−9^ per bp per year [14,15]. Because sequencing pedigrees is a much more direct and, in principle, definitive approach to learn about mutation, these new rate estimates have been widely adopted. They have led to a reappraisal of the chronology of human evolution, suggesting in particular that populations split longer ago than previously believed (e.g., [14,16]). Extrapolating farther back in time becomes problematic, however, as pedigree-based estimates imply split times with other primates that are older than is compatible with the fossil record, at least as currently interpreted [17–21]. One possible solution, suggested by Scally and Durbin (2012) [14] as well as others, is that yearly mutation rates have decreased towards the present, consistent with the “hominoid rate slowdown” observed in phylogenetic data [22–24].

As we discuss, changes in the yearly mutation rate over the course of human evolution are not only plausible but follow from first principles. The expected number of de novo mutations inherited by a child depends on paternal (and, to a lesser extent, maternal) ages at puberty and reproduction [25–27], traits that differ markedly among extant primates [18,28,29]. Because these traits evolve, there is no fixed mutation rate per generation and almost certainly no fixed mutation rate per year. An important implication is that the use of mutations to date evolutionary events requires a precise characterization of how germline mutations accumulate in development in males and females and across species. We argue that this knowledge is still elusive and that, as a result, it remains unclear how to set the evolutionary clock. For recent time depths, however, a complementary approach from the study of ancient DNA samples may offer a solution.

In discussing these points, we focus almost exclusively on humans, in part because, in other species studied to date, estimates of de novo mutation rates are instead higher than substitution rates, and the underlying reasons are likely distinct [30–33]. Likewise, we do not discuss mutation rates estimates for mitochondrial DNA; the sources of mutations, complete linkage, and selection pressures make the evolutionary dynamics of this one locus quite distinct from those of the nuclear genome, and, indeed, the discrepancy there, too, is opposite [34,35]. Moreover, we concentrate on the rate of single nucleotide substitutions in autosomes; for other types of mutations and a discussion of variation in mutation rates along the genome, see [25,36–38].

## The Puzzle

Heritable mutations stem from accidental changes to the genome that occur in the development of the germline and production of egg and sperm. A natural definition of the germline mutation rate “per generation” is, therefore, the rate at which differences arise between the genome of a newly formed zygote and the gametes that it eventually produces. While this quantity cannot be readily measured, it has recently become possible to estimate something highly related: the number of mutations seen in the genome of an offspring’s soma but absent from the parents’ [39] (henceforth *μ*_*G*_). At least a dozen whole genome studies have applied this approach, resequencing parents and offspring, usually in trios. They reported estimates of *μ*_*G*_ on the order of 10^−8^ per bp (Table 1).

**Table 1:** Estimates of mutation rates from pedigree studies.

Study	Reported mutation rate per bp per generation (x10^-8^)	Mean paternal age in study (in years)	Mutation rate at paternal age of 30 years (x10^-8^)^†^	Paternal age effect reported as the increase in number of mutations for each year of father’s age	Callable genome in Gb (reported false negative rate [FNR] in %)	Sample size (number of trios)	Mean sequence coverage	Fraction of CpG transitions^¶^
**Chimpanzee pedigree study:**								
Venn 2014^#^ [40]	1.20	24.3^a^	1.51	3.00	2.4 (13.4^b^)	6	34.4^b^	0.239 (0.183–0.296)
**Human pedigree studies:**								
Roach 2010^§^^,^^c^ [39]	1.10 (0.68–1.70)	—	—	—	1.8 (5.0)	2	61.3^b^	0.178 (0.037–0.320)
Conrad 2011 (CEU) [41]	1.17 (0.88–1.62)	—	—	—	2.5 (5.0)	1	29.3	0.146 (0.046–0.246)
Conrad 2011 (YRI) [41]	0.97 (0.67–1.34)	—	—	—	2.5 (3.5)	1	29.2	0.114 (0.009–0.220)
Campbell 2012 [42]	0.96 (0.82–1.09)	26.3	—	—	2.2 (1.7)^b^	5	13.0	0.165 (0.110–0.220)
Kong 2012 [15]	1.20	29.7	1.21	2.01	2.6 (2.0)	78	30.0	0.173 (0.163–0.184)
Michaelson 2012 [43]	1.00	33.6	0.93	1.02	2.8^e^ (9.5)	10	30.0	0.128 (0.099–0.156)
Jiang 2013 [44]	—	34.4^e^	—	1.50	—	32	30.0	0.162 (0.146–0.177)
Francioli 2015 [38]	—	29.4	—	1.20^*^	2.1 (31.1)	250	13.0	0.165 (0.158–0.172)
Besenbacher 2015 [45]	1.27 (1.16–1.38)	28.4	1.3^d^	2.00	—	10	50.0	0.201 (0.166–0.236)
Rahbari 2015^c^ [46]	1.28 (1.13–1.43)	29.8	1.29	2.87 (1.46–3.65)^f^	2.5 (—)	12	24.7	0.210 (0.180–0.240)
Yuen 2015^§^^,^^g^^,^^c^ [47]	1.18^h^	34.1	1.08	1.19^*^	2.5 (8.0^i^)	140	56.0	0.159 (0.151–0.167)
Wong 2016^§^^,^^‡^ [48]	1.05	33.4	0.95	0.92	1.6 (13.0)	693	60.0	0.131 (0.127–0.135)
Goldmann 2016^§^^,^ ^‡^ [49]	—	33.7	—	0.91	—	816	60.0	0.179 (0.175–0.182)

—Not available.

^§^ - Denotes studies that used Complete Genomics technology for sequencing, as most were based on Illumina sequencing.

^†^ - Estimated assuming linearity and using the reported paternal age effect, accounting for the length of the callable genome. Specifically, we used: $\hat{\mu_{g}}+\left(\frac{\hat{m}(30-P)}{2N}\right),$ where $\hat{\mu_{g}}$ is the estimated mutation rate per bp per generation reported in the study, $\hat{m}$ is the estimated slope for the paternal age effect, *P* is the mean paternal age in the study, and *N* is the length of the callable genome in base pairs.

^*^—A paternal age effect is not reported in paper but estimated by Poisson regression on counts of autosomal de novo mutations.

^¶^ - CG dinucleotides (CpG) fraction based on autosomal mutations and binomial 95% CI shown. When possible, we relied on validated mutations. However, in some studies, only a small fraction of mutations were validated, and, hence, we used all putative de novo mutations.

^#^—This study includes one multigenerational pedigree.

^‡^ - These studies found a significant maternal age effect, which might lead to lower estimates of the paternal age effect (if parental ages are correlated).

a—This is the estimated mean age of reproduction of male chimpanzees in the wild, not the age of the actual individuals studied (18.9 for males and 15.0 for females).

b—Refers to average, the estimate varies across individuals or families in the study.

c—Includes siblings.

d—Based on visual inspection of slope reported in the study.

e—Not reported in the article, based on personal communication.

f—Based on validated de novo mutation counts and extrapolated to a surveyed genome size of 3 gigabases (Gb).

g—All estimates are based on the subset of the 140 non- lymphoblast-derived cell lines (LCL) samples.

h—Not reported in the article; based on the number of de novo mutations reported in the study and an estimated denominator (2.5 Gb, personal communication).

i—Average of the estimates based on two cohorts.

Although the trio studies were primarily conducted to identify de novo disease mutations, they also inform our understanding of the chronology of primate evolution. Assuming that changes to the genome are neutral, the expected sequence divergence (that is, the expected number of substitutions per bp) between a pair of species, *d*, equals 2*μt*, where *μ* is the mutation rate and *t* is the average time to a common ancestor (i.e., divergence time). Thus, given an estimate of *μ* and orthologous sequences from more than one species, an estimate of *t* can be obtained from *d*/2*μ*. In practice, researchers are interested in an estimate of *t* in years, not generations, and therefore require an estimate of the yearly mutation rate, ${\hat{\mu }}_{y}$. To obtain it, common practice has been to divide the ${\hat{\mu }}_{G}$ obtained from sequencing of parents and children by a typical age of reproduction (i.e., an estimate of the generation time) [14]. Doing so yields ${\hat{\mu }}_{y}$ ≈ 0.5 x 10^−9^ per bp [14,50].

Taken at face value, this mutation rate suggests that African and non-African populations split over 100,000 years [14,16] and a human-chimpanzee divergence time of 12 million years ago (Mya) (for a human–chimpanzee average nucleotide divergence of 1.2% at putatively neutral sites) [10,14,17]. These estimates are older than previously believed, but not necessarily at odds with the existing—and very limited—paleontological evidence for Homininae [16,26,51]. More clearly problematic are the divergence times that are obtained for humans and orangutans or humans and OWMs. As an illustration, using whole genome divergence estimates for putatively neutral sites [10] suggests a human–orangutan divergence time of 31 Mya and human–OWM divergence time of 62 Mya. These estimates are implausibly old, implying a human–orangutan divergence well into the Oligocene and OWM–hominoid divergence well into or beyond the Eocene. Thus, the yearly mutation rates obtained from pedigrees seem to suggest dates that are too ancient to be readily reconciled with the current understanding of the fossil record [51,52].

Another way of viewing the same problem is to compare values of ${\hat{\mu }}_{y}$ obtained from resequencing pedigrees to those obtained from divergence levels among primates, given estimates of divergence times *t* based on the fossil record. Such estimates of *t* are highly indirect, in part because the fossil record is sparse and in part because relying on fossils with derived traits provides only a lower bound for when the species split [19,20]. A further complication is that, for closely related species, *t* reflects the time since the species split as well as the average time to the common ancestor in the ancestral population, which can be substantial [17,53]. Notably, for humans and chimpanzees, the divergence time *t* is thought to be at least 2 million years older than the split time, and possibly much more [17,21,54]. Thus, this approach is mired in uncertainty. Nonetheless, until recently, the consensus in the field has been to use $\hat{t}$ values of 6–7.5 Mya for humans and chimpanzees [9,55], 15–20 Mya for humans and orangutans [56], and 25–35 Mya for humans and OWMs [23,56,57]. Assuming these values and solving ${\hat{\mu }}_{y}=d/2\hat{t}$ suggests a mutation rate of 10^−9^ per year, more than 2-fold higher than what is obtained from pedigree-based estimates (Table 1). In other words, accepted divergence times suggest that substitutions accumulate faster than they should based on mutation estimates from human pedigrees.

## Could This Puzzle Be Resolved by Purifying Selection or Biased Gene Conversion?

The equation of mutation and substitution rates is valid only under neutrality. The substitution rate of a population can be factored into two components: the rate at which mutations arise in the population and the probability that a mutation is eventually fixed in the population. When changes are neutral, larger populations experience a greater input of mutations, but exactly counterbalancing this effect is a smaller probability of fixation for each mutation. When natural selection is operating, however, the probability of fixation deviates from the neutral expectation, so the substitution rates at sites under selection are not expected to equal the mutation rate.

To minimize this problem, researchers have focused on putatively neutral regions of the genome when estimating divergence levels (e.g., pseudogenes or genomes with genic and conserved regions excluded) [9,10]. This filtering process is imperfect, however, as putatively neutral regions likely include sites under some degree of natural selection. The net effect should be to decrease the substitution rate relative to the mutation rate, because deleterious mutations that contribute to the count of de novo mutations in pedigrees will not reach fixation (and beneficial alleles are exceedingly rare). In other words, selection should lead to lower substitution rates than mutation rates [34] and likely provides at least a partial explanation for the patterns observed in many taxa (including *Drosophila*, *Arabidopsis*, and *Caenorhabditis*), as well as for the human mitochondrial DNA [31,34]. In contrast, selection only exacerbates the puzzle of why estimated substitution rates are higher than estimated mutation rates in the human nuclear genome.

In addition to selection, the fixation probability can also be affected by GC-biased gene conversion (BGC). This process preferentially resolves mismatches in heteroduplex DNA arising from meiotic recombination in favor of strong alleles (C or G) over weak alleles (A or T), leading to an increased fixation probability of mutations from A/T to C/G and a decreased probability of C/G to A/T mutations relative to neutrality. Although clear evidence for BGC has been observed both in mammalian substitution patterns and in human pedigree data [58,59], the net impact on genome-wide substitution rates remains unclear. Moreover, a similar discrepancy between pedigree and phylogenetic estimates of mutation remains when focusing only on the subset of sites not subject to BGC [10,15]. Thus, this process is unlikely to help reconcile the estimates either.

**Fig 1:**
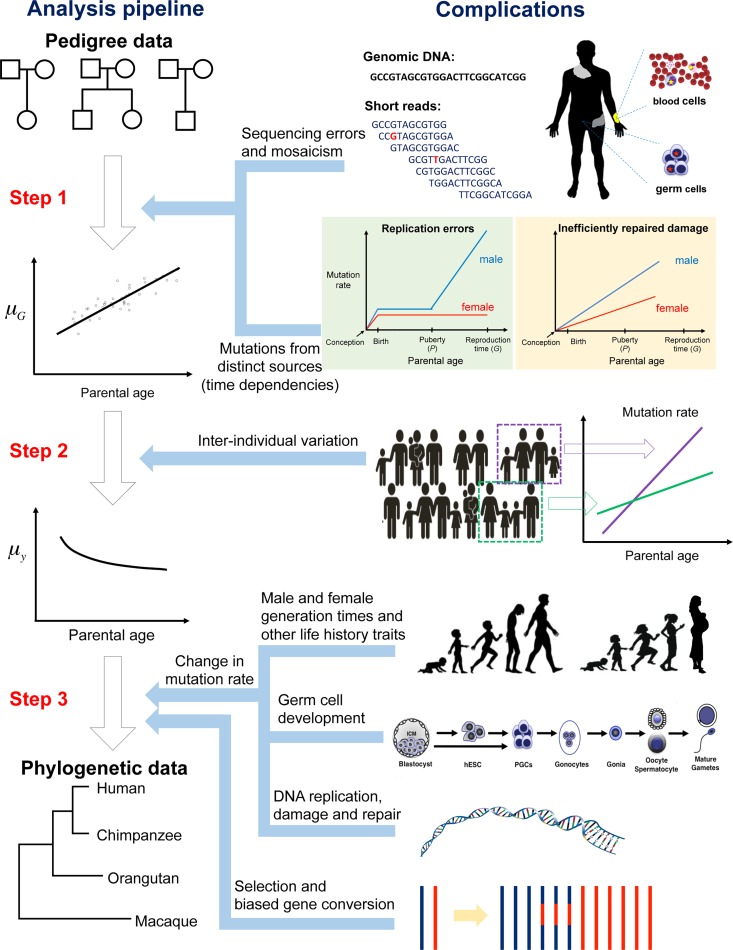
The many steps involved in the conversion of mutation rate estimates from pedigree studies into yearly substitution rates.

With no obvious explanation at hand, the surprisingly low mutation rates estimated from pedigrees have led to considerable discussion about whether our understanding of primate evolution is simply incorrect, and divergence times are much older than believed. We contend that in some ways this reevaluation is premature. Indeed, although our current understanding of the primate fossil record could be inaccurate, there is underappreciated complexity in the conversion of mutation rates from pedigrees into mutation rates per year and its translation into substitution rates, which remains to be resolved. We try to unpack this complexity by discussing each step in turn: (1) what it is we are truly estimating from resequencing pedigrees; (2) what we have learned to date and what we have yet to understand; and (3) how to translate the mutation rates into evolutionary dates (Fig 1).

## Step 1: What Exactly Is Being Estimated from Human Pedigrees?

Human pedigree studies have relied primarily on blood samples from trios by identifying mutations present in ~50% of reads in the child but absent in both parents. A mutation rate is obtained by dividing the count of mutations by the number of base pairs for which there was complete power to identify de novo mutations or, equivalently, dividing it by the genome length, adjusting for power at a typical position in the genome (assuming mutation rates in inaccessible regions of the genome are similar to those in surveyed regions).

Because the mutation rate is so low (~10^−8^ per bp per generation), it is challenging to reliably identify de novo mutations using current sequencing technologies, given the presence of cryptic copy number variation, alignment uncertainty, and other confounders [60,61]. Detection pipelines, therefore, have high false discovery rates, and a stringent set of filters on sequence complexity, read depth, and allelic balance of the reads has to be applied to weed out spurious mutations [62]. This aggressive filtering process substantially increases specificity but decreases the number of sites at which mutations can be detected, so the false negative rate has to be carefully assessed for any given set of filters.

**Fig 2:**
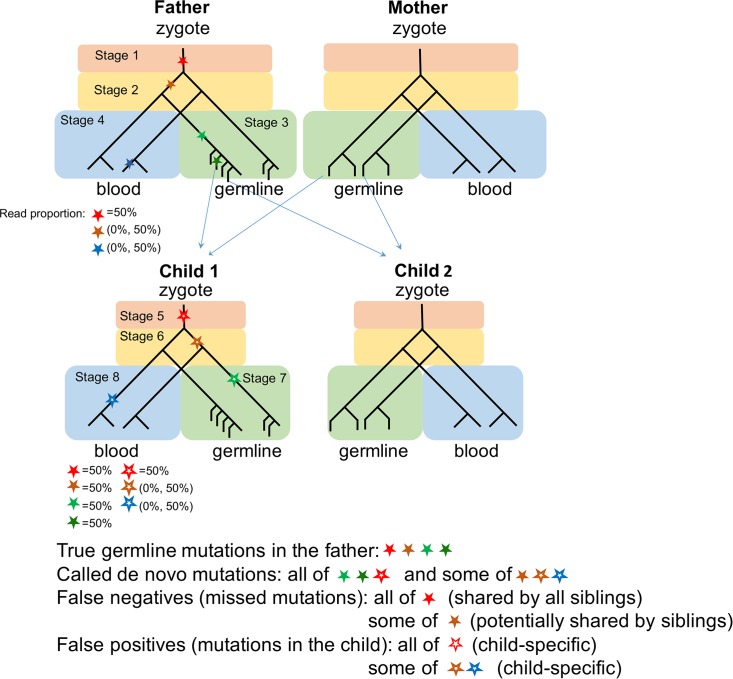
Schematic illustration of mutations occurring during embryonic development and gametogenesis. For simplicity, we show only mutations that arose in the father and one offspring (child 1). Stars represent mutations that originate in different stages of embryogenesis and gametogenesis of the father and the offspring; solid stars are mutations that arise in the father, and hollow stars are those that occur in the offspring. Shown below each individual are the expected frequencies of the labeled mutations in his or her blood sample. Red, brown, and green stars are heritable and should be included in an estimate of germline mutation rates, whereas blue stars are somatic mutations present only in blood samples, which should be excluded. The detection of mutations that are mosaic in both soma and germline strongly suggests that, in the cell lineage tree of human development, soma and germline are not reciprocally monophyletic [46,64]. The standard pipelines require allelic balance in the child and no (or very low) read depths in the parents, leading to inclusion of some postzygotic mutations in the child and exclusion of a fraction of germline mutations in the parents. The two effects partially balance, so the overall mutation rate is unlikely to be greatly biased. However, there is a tendency to detect child-specific mutations and to miss ones shared among siblings. As a consequence, the mutation rates during early development are likely underestimated, with potentially important practical implications for predictions of recurrence risk of diseases caused by de novo mutations.

An additional complication is “mosaicism,” that is, the presence of two or more genotypes in a given population of cells. When calculating the mutation rate per generation, any mutation accumulated in a germline cycle from zygote to zygote should be included regardless of the stage at which it occurred (Fig 2, solid stars). The difficulty is that neither the parents nor the offspring are sampled as zygotes; instead, blood samples are used. In these somatic samples, some of the mutations detected will have arisen during the development process of the child and should not be counted towards germline mutations in the parents (Fig 2 hollow stars; [63]). Moreover, when multiple reads support the alternative allele in the blood of the parent, it is unclear whether the mutation is mosaic and present at high frequency or truly constitutional (i.e., heterozygous in all cells). Standard filtering process requires there to be a balanced number of reads carrying both alleles in the child and no (or very few) reads with the alternative allele in both parents. These criteria will lead to the inclusion of some postzygotic mutations that arose in the child (in germline and/or soma) and the exclusion of a fraction of true germline mutations in the parents (especially those that arose in early development stages) (Fig 2).

Given the current de novo mutation detection pipelines, the presence of mosaicism therefore leads to two complications: (1) it may lead to a systematic bias in the estimate of the germline mutation rate per generation, and (2) it may distort estimates of per cell division mutation rates in different stages of germline development due to misassignment of the detected mutations to different stages. Current evidence suggests that the first concern is a minor one, both because false negatives and positives are expected to balance each other out to some extent, and because, in practice, similar estimates are obtained when considering transmissions in trio studies and when analyzing autozygous segments that descend from a common ancestor multiple generations back (i.e., in which mutations that arose in two or more complete germline cycles are captured) (Table 1) [42,65]. Nonetheless, the current filtering criteria will lead to an underestimate of mosaicism levels and could cloud our understanding of the germline mutational process, impacting the accuracy of predictions about the recurrence risk of diseases caused by de novo mutations (see Fig 2) [46,66,67].

In addition to these technical considerations, there are conceptual subtleties in interpreting the mutation rate estimates from pedigree studies. As expected a priori and from earlier studies of disease incidences in children [27,68], all large pedigree studies published to date have reported an effect of the age of the father on the total number of de novo mutations inherited by a child (Table 1). Moreover, the increase in the total number of mutations is well approximated by a line [15] (a phenomenon distinct from the few well-studied mutations, such as fibroblast growth factor receptor 2, which occur during spermatogonial stem cell divisions and lead to clonal expansions, and for which the increase in frequency with paternal age is closer to exponential [69–71]). Because spermatogenesis occurs continuously after the onset of puberty, the number of replication-driven mutations inherited by a child is expected to depend on paternal age—more precisely, on the age at which the father enters puberty, his rate of spermatogonial stem cell divisions, and age at reproduction [25,72]. Therefore, the observation that the number of mutations increases linearly with paternal age is consistent with a fixed rate of cell division after puberty and a constant rate of mutation per cell division during spermatogenesis. In contrast, oocytogenesis is completed by the birth of the future mother, so the number of replication-driven mutations inherited by an offspring should be independent of maternal age [73]. For the subset of mutations that do not stem from mistakes during replication—mutations that arise from DNA damage and are poorly repaired, for example—there may be a dependence on maternal age as well, if damage accumulates in oocytes [74]. Interestingly, recent studies report that a maternal age effect is also present, potentially supporting the existence of a nonreplicative source of germline mutations [48,49,75]. In any case, what is clear is that the number of de novo mutations in a child is a function of the age of the father at conception and, to a lesser extent, that of the mother, so values obtained from pedigree studies are estimates of mutation rate at given mean paternal (and maternal) ages of the sampled families.

Another complication is that distinct types of mutations may differ in their accrual rates with age, depending on their sources and repair rates over ontogenesis [74,76]. For instance, transitions at methylated CG dinucleotides (CpG) sites are thought to occur primarily by spontaneous deamination; beyond this example, the DNA molecule is known to be subject to a large number of chemical assaults from normal cellular metabolism and additional environmental agents [77,78]. Although the relative contribution of germline mutations from different sources is unclear, their accrual rates with parental age are unlikely to be identical [49]. Therefore, the mutation rate estimated from pedigree studies is the composite of distinct mutational processes that have distinct dependencies on age and sex [49], making the time-dependency of the overall mutation rate harder to interpret (Fig 1).

With these considerations in mind, what have we learned to date? All large-scale pedigree studies report similar mutation rates per generation, a strong male bias in mutation, and a paternal age effect. On closer inspection, however, their parameter estimates are not consistent. To illustrate this point, we report the estimated mutation rate at paternal age of 30 years, which differs by as much as 40% across studies (Table 1). Given the relatively small sample sizes, some uncertainty is expected from sampling error alone. However, differences in sequencing technology, coverage depth, and choice of filters are also likely to be playing a role. As one illustration, the fraction of mutations that involve transitions from CpG sites differs significantly among studies, from 11% to >20% (chi-square test, *p* < 10^−8^, considering studies with a sample size of at least 5). Although biological differences cannot be ruled out, at least some of this variation appears to be due to whether the studies excluded mutations present in dbSNP [79] (because the authors reasoned that a sequencing error is a more likely explanation than a recurrent mutation). As databases become larger, this step increasingly leads to the exclusion of true mutations [80], with a disproportionate effect on CpG transitions, which are more mutable [45].

**Fig 3:**
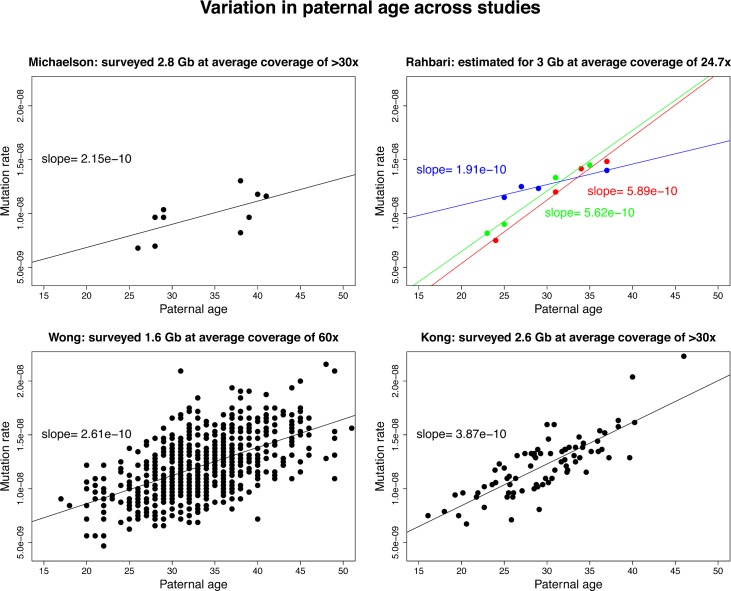
Variation in the estimated paternal age effect for autosomes. We plot the de novo mutation rate as a function of the paternal age at conception of the child. The rate was obtained from the reported counts of de novo mutations divided by the fraction of the genome assayed in each study (shown in the title of each subplot, along with the mean sequence coverage per individual). The solid line denotes the fitted slope (i.e., the increase in the mutation rate for each additional year of father’s age). Following the approach of Rahbari et al. 2015 [46], for their study, we used the corrected counts of de novo mutations, which are extrapolated to a genome length of 3 Gb (thereby assuming the mutation rate in the inaccessible regions of the genome is the same as that in surveyed regions). The three colors used in this plot denote the three different families that were studied: blue, family 244; green, family 603; and red, family 569.

Among studies, there is also 3-fold variation in the estimated strength of the paternal age effect (Table 1), which remains significant after accounting for the fraction of the genome surveyed for mutation (Fig 3). In principle, differences in the paternal age effect among studies could reflect true biological differences. For instance, a recent study of three larger pedigree families reported that the fathers differed markedly in their paternal age effects (Fig 3) [46]. If, indeed, fathers differ in the strength of their paternal age effect, then when a single line is fit to data from their offspring, the resulting slope could differ, possibly substantially, from the average slope [25,74]. As the sample size increases, however, the estimated strength of paternal age effect should approach the population mean value, so the observed differences across large studies remain unexplained.

In summary, although pedigree-based approaches are more direct and, in principle, straightforward, they have not yet provided a definitive answer about the mutation rate at any given paternal and maternal age, let alone a precise characterization of how mutations of different sources accumulate over ontogeny in males and females.

## Step 2: How to Obtain a Yearly De Novo Mutation Rate?

Even if the germline mutation rate per generation, *μ*_*G*_, were known exactly, strong assumptions would be required to translate the per generation mutation rates of the sampled families into a yearly rate. Common practice has been to obtain a yearly mutation rate by dividing the mutation rate estimated from all the children by the typical age at reproduction (i.e., setting ${\hat{\mu }}_{y}={\hat{\mu }}_{G}/\bar{G}$, where $\bar{G}$ is an estimate of the generation time in the population or the average age of the parents in the study). This practice implicitly ignores differences in reproduction ages across studies or between the individuals studied and the general population [81]. When such differences exist (Table 1), the values of ${\hat{\mu }}_{y}$ are only comparable if the same $\bar{G}$ is used and the numerator is replaced by ${\hat{\mu }}_{y}(\bar{G})$, the expected (per generation) mutation rate at a particular age $\bar{G}$. Moreover, if *μ*_*y*_ is not independent of *G*, as suggested by modeling and (limited) available data in humans [25], then it is also important to ask whether the mean parental age for the (predominantly European) samples is representative of the human species, when it is known that ages at reproduction differ substantially across populations [81].

Also complicating matters are possible differences among fathers in the onset of puberty or the strength of their paternal age effect. We know that there is heritable variation among humans (from the same population) in the onset of puberty [82], and there are hints of differences in rate of spermatogonial stem cell divisions or per cell division mutation rates across males [46]. If substantial differences in these factors also exist across human populations, the relationship between *μ*_*G*_ and *G* will vary among them. Therefore, even when the sample size is large and variation in *G* taken into account, the estimated yearly mutation rate from ${\hat{\mu }}_{y}={\hat{\mu }}_{G}/\bar{G}$ may only be representative of the population(s) under study.

## Step 3: How to Relate *μ* to the Substitution Rate Expected over Evolutionary Time?

### Changes in Life History and Reproductive Traits

Mammalian species vary over 3-fold in yearly substitution rates, indicating that the yearly mutation rates change over time [83–85]. In primates, in particular, 35%–65% variation is seen in substitution rates across apes and monkeys [10,11]. The cause of variation in substitution rates was long hypothesized to be a “generation time effect,” whereby younger mean ages of reproduction—i.e., shorter generation times—lead to more cell divisions per unit time and, hence, higher rates of replication-driven mutations [23,86–88]. Support for this claim comes from phylogenetic analyses of mammals, in which reproductive span is the strongest predictor of mutation rates per year among various correlated traits considered, including onset of puberty, body size, metabolic rate, longevity (notably for mitochondrial DNA), and sperm competition [85,89,90].

As we have discussed, a dependence of yearly mutation rates on generation times is expected from what is known about mammalian sperm and egg production. Thus, to accurately convert mutation rates per generation into expected substitution rates per year, changes in the generation time over evolution need to be taken into account. Doing so requires knowledge of numerous parameters that are currently uncertain or simply unknown. A solvable problem is that the conversion depends on the precise dependence of *μ*_*G*_ on parental ages [25], about which there remains considerable uncertainty (Fig 3). A thornier issue is that the yearly substitution rate depends not only on the sex-averaged generation time, but also on the mean ages at reproduction for males and females separately. The reason being that, in males, the germline mutation rate depends more strongly on reproductive age than it does in females; thus, for the same average parental age, de novo mutation rates are much lower in a child born to a young father and an old mother than in a child born to an old father and a young mother. As a result, changing the ratio of male-to-female generation times can have substantial effects on the yearly mutation rate, even when the average remains fixed: for example, a range of ratios from 0.92 to 1.26, as observed in extant hominines, could lead to up to 10% difference in *μ*_*y*_ and thus introduce uncertainty in phylogenetic dating [26].

Beyond the effect of generation times, the yearly mutation rate will vary with any change in life history traits (e.g., the age at puberty) and germ line developmental process (e.g., the number of cell divisions in each development stage). We know that, among extant primates, the onset of puberty differs substantially, from ~1 year in marmosets to 6–13 years in apes [28], as does the length of spermatogonial stem cell divisions [91]. Thus, life history traits can and have evolved across primates. This evolution introduces additional uncertainty in the yearly mutation rate expected at any point in the past [26]. Moreover, these factors influence *μ*_*y*_ in intertwined ways, so it is important to consider their joint effects [10,26].

### Changes in the Mutation Process

Thus far, we have only discussed sources of changes in the yearly mutation rate due to development and life history, but another layer of evolution occurs at cellular level, in terms of mutational processes of DNA [92–94]. Could the rates of replication error, DNA damage, or DNA repair have evolved over millions or even thousands of years? Two recent studies have compared the spectra of rare segregating variants among human populations and found enrichment of specific mutational signatures in certain populations [94,95]. For instance, Europeans show an increased rate of a TCC → TTC mutation relative to African or Asian population samples [94]. Additionally, a recent study of autozygous segments in a Pakistani population found some germline mutations to occur at significantly different frequencies than in samples of European ancestry [65]. This observation raises the possibility of recent evolutionary changes in the mutation process itself.

Although a change in mutation rates of a specific mutation type is parsimoniously explained by a change in the damage or repair rates, modeling suggests that, even in the absence of such changes, life history traits alone could shift the relative contributions of mutations of different sources [74]. As one example, CpG transitions appear to be more clocklike across species than do other types of mutations (possibly due to a weaker dependence on life history traits) [10,84], and, accordingly, the proportion of substitutions due to CpG transitions varies across species [10]. As another example, an increase in paternal age leads not only to an increase in the total germline mutation rate but also to a slight increase in the proportion of mutations in genic regions [38], which should lead to shifts in the mutation spectrum [49]. More generally, it remains highly unclear to what extent differences in mutation rates across populations or species can be attributed to changes in life history and behavior, in the development and renewal of germ cells, in genetic modifiers of mutation (such as enzymes involved in DNA replication and repair) [92,96], or in the environment (such as temperature or the concentration of external mutagens) [97,98].

## Next Steps

To predict the rate at which substitutions will accumulate from pedigree data is not as straightforward as it may seem. The main difficulty is that, although in some contexts, it is a useful concept, there is in fact no such thing as a mutation rate “per generation”—all that exists is a mean mutation rate for a given set of paternal and maternal life history traits, including ages at puberty and reproduction. These traits are variable among closely related primates [28,29], and heritable variation is seen even among humans [81,82]. Therefore, primate species are expected to differ substantially in both the per generation mutation rate and the yearly mutation rate (e.g., see Table S9 in [26]).

Indeed, phylogenetic analyses show that, over millions of years, substitution rates vary >60% among distantly related primates [10]. The variation in substitution rates across primates and mammals appears to be smaller than that predicted from life history traits in extant species, however [10,26,30]. A likely reason is that, throughout much of their evolutionary past, the lineages had similar life histories. Direct surveys of de novo mutation rates in nonhuman primates are therefore needed to test whether the present-day mutation rates are more or less similar to those predicted based on life history traits alone.

So far, the only direct estimate of mutation rate in a nonhuman primate is based on one three-generation pedigree of chimpanzees [40]. The point estimate of the mutation rate at age 30 is higher in chimpanzees than in humans (Table 1), qualitatively consistent with an earlier onset of puberty and faster rate of spermatogenesis [28,91]. Given the differences in detection pipelines, random sampling error, and potential intra-species variation, however, these results are still tentative. Both inter- and intraspecies variation in mutation rates need to be further characterized in primates.

If mutation rates turn out to vary substantially across species, it will be interesting to examine whether they are well predicted by typical ages at puberty and reproduction. A positive correlation of mutation rate per generation and generation time across species would imply that, over evolutionary timescales, the yearly mutation rate is less variable than the mutation rate per generation, contrary to what is usually assumed (e.g., [87,99]).

If, on the other hand, despite clear differences in life history traits, the per generation mutation rate across mammals turns out to be relatively constant, strong stabilizing selection or developmental constraint must have shaped the evolution of mutation rates. A hint in that direction is provided by recent estimates of mutation rates in mice, whose generation times are on the order of months rather than decades as in apes, and yet whose mutation rate per generation is only about half that of humans [30,100]. It would follow that species with longer generation times will have lower yearly mutation rates, providing more direct support for the “generation time effect” than can be obtained from phylogenetic evidence [23,83,87].

That yearly mutation rates are expected to be unsteady poses difficulties for the use of substitutions to date evolutionary events. One solution is to explicitly model the changes in life history traits over the course of primate evolution and to study their impact on substitution rates. To this end, Amster and Sella (2016) [26] proposed a model that estimates divergence and split times across species, accounting for differences in sex-specific life history and reproductive traits. A next step will be to extend their model to consider replicative and nonreplicative mutations separately. In addition, as more reliable estimates of mutational parameters become available from pedigree studies of humans and nonhuman primates, models will need to be revised to account for differences in cell division rates and possible differences in repair rates. Unfortunately, however, some uncertainty will remain due to lack of knowledge about life history traits in ancestral lineages.

An alternative might be to use only CpG transitions for dating. This solution is based on the observation that CpG transitions accumulate in a quasi-clocklike manner across primates [10,84], as well as across human populations [94]. Puzzlingly, however, in human pedigree data, there is no detectable difference between the effects of paternal age on CpG transitions and on other types of mutations [15,46], suggesting that CpG transitions are no more clocklike. In that regard, it will be highly relevant to compare accrual rates of CpG transitions in large pedigree studies from multiple primate species.

In addition to the use of pedigree studies, two other types of approaches have been introduced recently to learn about mutation rates. The first is a set of ingenious methods that use population genetic modeling to estimate mutation rates based on segments of the genome inherited from a distant common ancestor [101,102]. Unfortunately, these methods rely on a number of other parameter estimates, including a demographic model (on which the times to the most common ancestor are based), fine-scale meiotic recombination rates, and, to obtain yearly rates, generation times. A related idea is to estimate mutation rates from autozygous segments that descend from a recent common ancestor that can be more reliably inferred. This approach presents a number of advantages, notably in minimizing the possible contribution of somatic mutations, but only provides a mutation rate averaged over both sexes and several generations [42,65].

It has also become possible recently to use reliably dated ancient DNA samples to estimate average yearly mutation rates over different evolutionary periods. Here, the divergence from an extant sample (e.g., human) to an outgroup (e.g., chimpanzee) is compared to what is seen between an ancient genome and the outgroup. The “missing sequence divergence” then provides an estimate of the average mutation rate per year over that timescale. Applied to archaic human samples from the past 50,000 years, this approach suggests yearly rates around 0.5 x 10^−9^ per bp [103]. As in the “tip calibration” approach for estimating the evolutionary rates using sequentially sampled virus genomes or ancient mitochondrial genomes [104], the study of many ancient human nuclear samples distributed across ancestral populations could, in principle, serve as “spike ins” for the evolutionary clock, allowing one to adjust for changes in rates over different time periods along a lineage.

Together, this combination of approaches will both inform us about how to reliably set the evolutionary clock and provide a first direct look at the evolution of mutation rates.

## Abbreviations

BGC: GC-biased gene conversion
bp: base pair
CpG: CG dinucleotides
FNR: False negative rate
Gb: gigabases
LCL: lymphoblast-derived cell lines
My: million years
Mya: million years ago
OWM: Old World Monkey
